# Intrahepatic Gossypiboma as a Complication of Cholecystectomy: A Case Report

**DOI:** 10.7759/cureus.77268

**Published:** 2025-01-11

**Authors:** Ana Rebeca Meza-Bosquez, Fernando A Núñez-Moreno, Vanessa Ortiz-Higareda

**Affiliations:** 1 Gastrointestinal Surgery, La Unidad Médica de Alta Especialidad (UMAE) Centro Médico Nacional Siglo XXI Hospital de Especialidades, Mexico City, MEX; 2 Neurosurgery, Carl Von Ossietzky Universität Oldenburg, Oldenburg, DEU

**Keywords:** conventional laparoscopic cholecystectomy, gossypiboma, hepatic abscess, surgical complication, textiloma

## Abstract

We present the case of a 75-year-old woman with a history of laparoscopic cholecystectomy, which was converted to an open procedure due to intraoperative hemorrhage. Two weeks later, she underwent reintervention to address a subhepatic hematoma. Over a follow-up period of 19 months, she remained symptomatic, experiencing persistent purulent discharge from the surgical drain site, mild fever, and abdominal pain. A CT scan revealed a gossypiboma, and following a failed surgical exploration, the patient was referred to a specialized center. A fourth surgical exploration identified an abscess in the abdominal wall and multiple abscesses within the liver, one of which contained an intrahepatic gossypiboma. Postoperative care involved a levofloxacin-based antibiotic regimen, leading to a favorable clinical outcome. This case represents an instance of a gossypiboma becoming fully internalized within the hepatic parenchyma following non-traumatic surgery.

## Introduction

Gossypibomas, also known as textilomas, are rare iatrogenic complications resulting from the unintentional retention of surgical textile materials within a patient's body. The true incidence of gossypiboma is challenging to determine due to legal implications, underreporting, and diagnostic difficulties in asymptomatic cases. However, prevalence has been estimated at approximately 0.004% in medical-legal claims [1]. Regional variations are observed: in Jordan, the incidence is reported to be around 0.198 per 1,000 surgeries [2], while in the United States, the incidence of retained surgical materials (RSM) is estimated at 0.356 per 1,000 surgeries, with textile-related cases accounting for 0.125 per 1,000 surgeries in a three-year retrospective analysis [3]. Unfortunately, no data are available from Latin America.

The abdominal region is the most frequently reported site for gossypibomas worldwide [2,4-6]. In patients with a history of cholecystectomy, gossypibomas are commonly located in the subhepatic region [7-9]. Their identification can be further complicated by their ability to migrate, eroding into adjacent organs [10-12]. A specific and rarer presentation is that of intrahepatic gossypibomas, where the retained surgical material becomes encapsulated within hepatic tissue. This phenomenon is typically associated with hepatic trauma involving a breach in the liver parenchyma and subsequent entrapment of foreign material [13]. In this report, we present a unique case of intrahepatic gossypiboma, where surgical gauze was encapsulated by hepatic parenchyma following a routine cholecystectomy.

## Case presentation

This report details the case of a 75-year-old woman with a history of laparoscopic cholecystectomy conducted on November 30, 2021. The procedure was converted to an open surgery due to intraoperative hemorrhage. Ten days postoperatively, she presented with fever and right upper quadrant pain. A second surgical intervention was performed on December 12, during which a residual hematoma near the cystic plate was identified and evacuated. A Penrose drain was placed in the subhepatic region, and the patient was discharged. The drain was removed 15 days later, and the patient was followed on an outpatient basis until June 2023 (19 months). During this period, she experienced persistent mild fever, mild right upper quadrant pain, and purulent discharge from the abdominal drain site despite undergoing multiple courses of antibiotics.

Due to the unfavorable clinical course, an abdominal computed tomography (CT) scan was performed, revealing the presence of a gossypiboma, which prompted the attending surgeon to undertake a third surgical exploration on June 15, 2023. Intraoperative findings revealed extensive adhesions in the upper right quadrant, with the colon and stomach attached to the hepatic flexure. However, no foreign body was identified. A biliary tract injury was suspected during adhesiolysis, leading to the patient's immediate referral to our hospital, a specialized center for hepatobiliary surgery.

Upon admission to our facility on the same day as surgery, the patient's body mass index (BMI) was 31.3, with a height of 1.44 m and a weight of 65 kg. Vital signs were recorded as follows: blood pressure, 130/90 mmHg; heart rate, 92 bpm; respiratory rate, 19 breaths per minute; temperature, 36.6°C; and oxygen saturation, 95% on room air. She was conscious and alert; only mild dehydration was observed in the oral mucosa, and a nasogastric tube was in place.

The cervical and thoracic regions showed no abnormalities. In the abdominal region, a 15-cm surgical wound in the right upper quadrant was noted. The wound edges were clean, with no signs of infection, and were held together with sutures. The abdomen was soft and depressible on palpation, with pain elicited upon firm pressure in the right hypochondrium. A Penrose drain in the right flank was observed, discharging purulent exudate, but there were no signs of peritonitis. The patient's extremities were unremarkable.

Fasting was initiated, and parenteral fluids were provided. Analgesia was achieved with intravenous ketorolac 30 mg every eight hours as needed for pain, and a new regimen of antibiotics was started with levofloxacin 500 mg every 24 hours. Laboratory tests were ordered, showing mild anemia, mild hypokalemia, and elevated lactate dehydrogenase levels (Table 1).

**Table 1:** Admission laboratory tests Na: sodium; K: potassium; Cl: chloride; HCT: hematocrit; ALT: alanine aminotransferase; AST: aspartate aminotransferase; LDH: lactate dehydrogenase

Parameters	Values	Normal Values
Glucose	108.7 mg/dL	70-105 mg/dL
Urea	17.3 mg/dL	15.2-52.3 mg/dL
Creatinine	0.6 mg/dL	0.57-1.11 mg/dL
Na	139 mEq/L	136-145 mEq/L
K	3.1 mEq/L	3.5-5.1 mEq/L
Cl	103.4 mEq/L	98-107 mEq/L
Hemoglobin	10 g/dL	13-18 g/dL
HCT	29.8%	42-53.6%
Platelets	334,000/mm^3^	150,000-450,000/mm^3^
Leukocytes	7600/mm^3^	4600-10200/mm^3^
Total bilirubin	0.71 mg/dL	0.2-1.2 mg/dL
Direct bilirubin	0.4 mg/dL	0-0.5 mg/dL
Indirect bilirubin	0.27 mg/dL	0-0.8 mg/dL
ALT	30.6 IU/L	0-55 IU/L
AST	94.8 IU/L	5-34 IU/L
LDH	352 IU/L	125-220 IU/L

**Figure 1:**
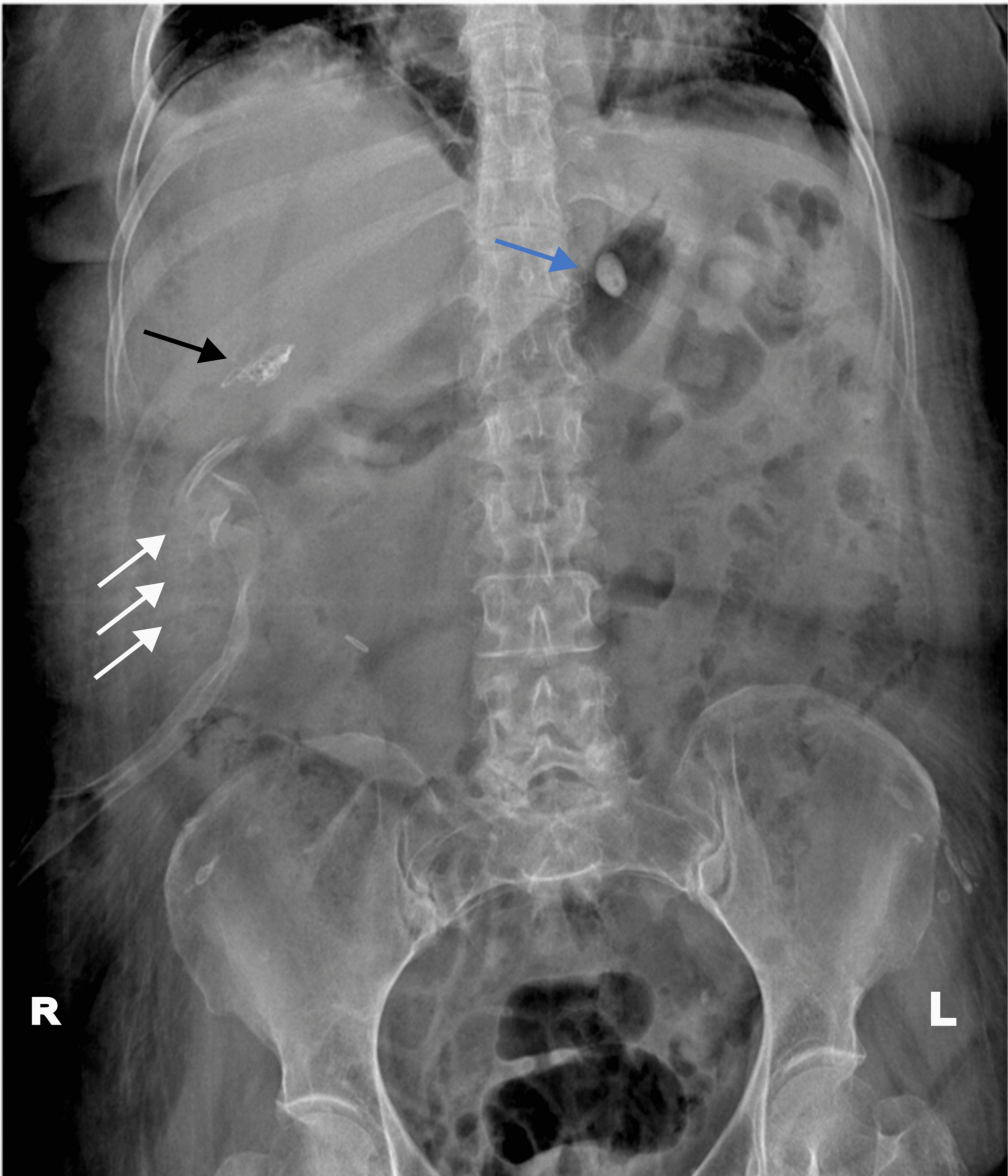
A simple abdominal X-ray in the decubitus position reveals multiple radiopacities located in the right hypochondrium. The white arrows denote the position of the surgical drain, while the black arrow indicates the presence of the gossypiboma within the hepatic shadow. The blue arrow points to another opacity seen in the stomach location that disappeared in subsequent imaging studies. Original image by the authors.

We began the diagnosis evaluation with a plain abdominal X-ray, which revealed the presence of a foreign body in the liver (segment V), suggestive of gossypiboma (Figure 1).

**Figure 2:**
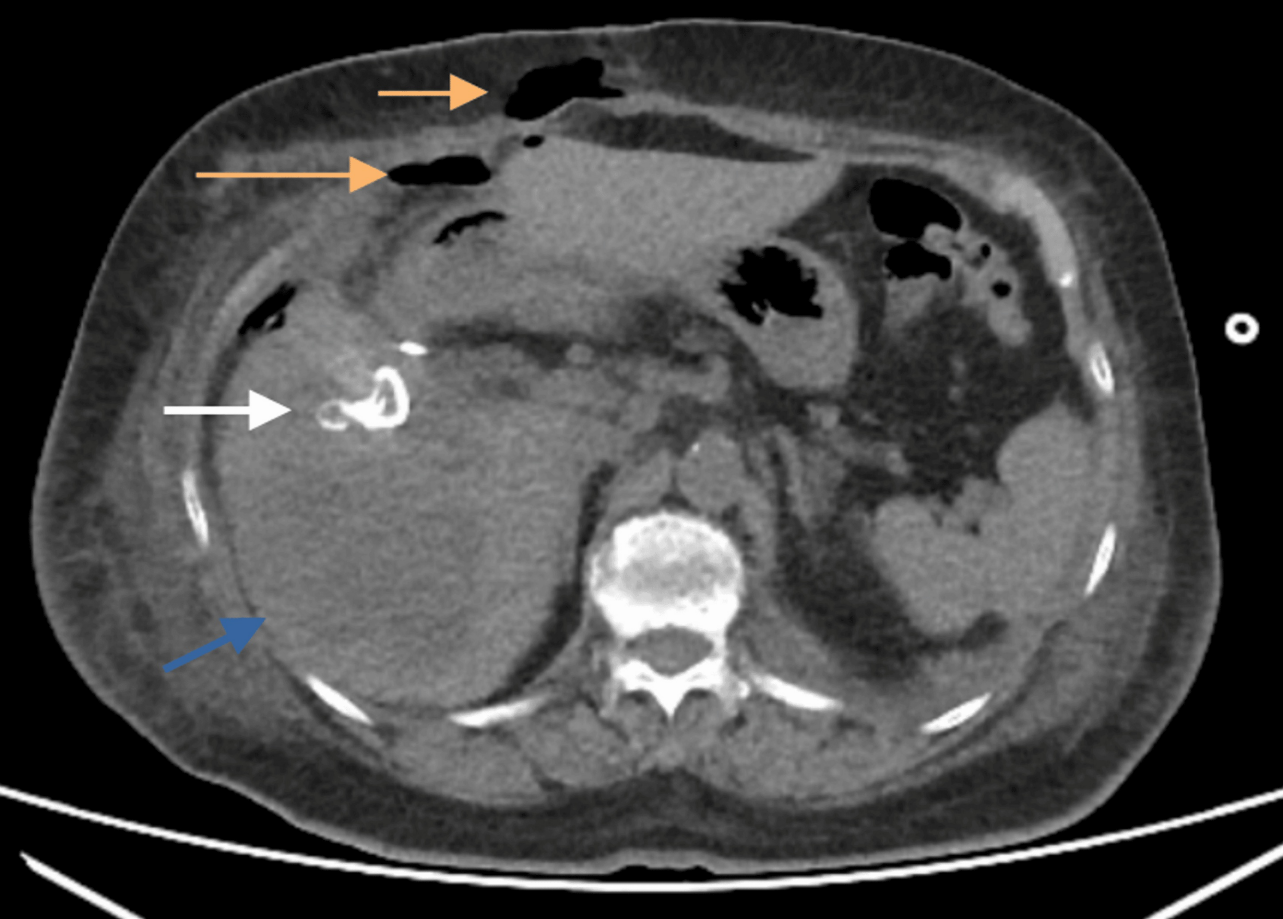
Abdominal CT showing a gossypiboma and post-surgical changes. The abdominal CT reveals the presence of a gossypiboma and changes related to the previous surgical intervention. Hypodense areas are observed in the abdominal wall and within the abdominal cavity (orange arrows), suggestive of air. In hepatic segment V, a hyperdense focal lesion is identified (white arrow), surrounded by heterogeneous zones within the liver parenchyma. These findings suggest an inflammatory reaction and abscess formation (blue arrow). Original image by the authors.

Following this, an abdominopelvic CT scan was performed to further investigate the location of the foreign object. The imaging revealed an occupying lesion in segments V, VI, and VII of the liver, with a multilocular appearance and hyperdense material noted in segment V (Figure 2). To provide additional detail, a biliary tract magnetic resonance imaging (MRI) scan was conducted. The MRI findings confirmed a Strasberg type C biliary tract injury, cholangiolar abscesses in segments V and VI, and a collection in the abdominal wall.

Based on the imaging findings, an exploratory laparotomy was performed under general anesthesia. The surgical findings included an abscess on the abdominal wall from which approximately 200 mL of hematopurulent material was drained. Upon entering the abdominal cavity, firm adhesions of the omentum and duodenum to the liver were observed.

**Figure 3:**
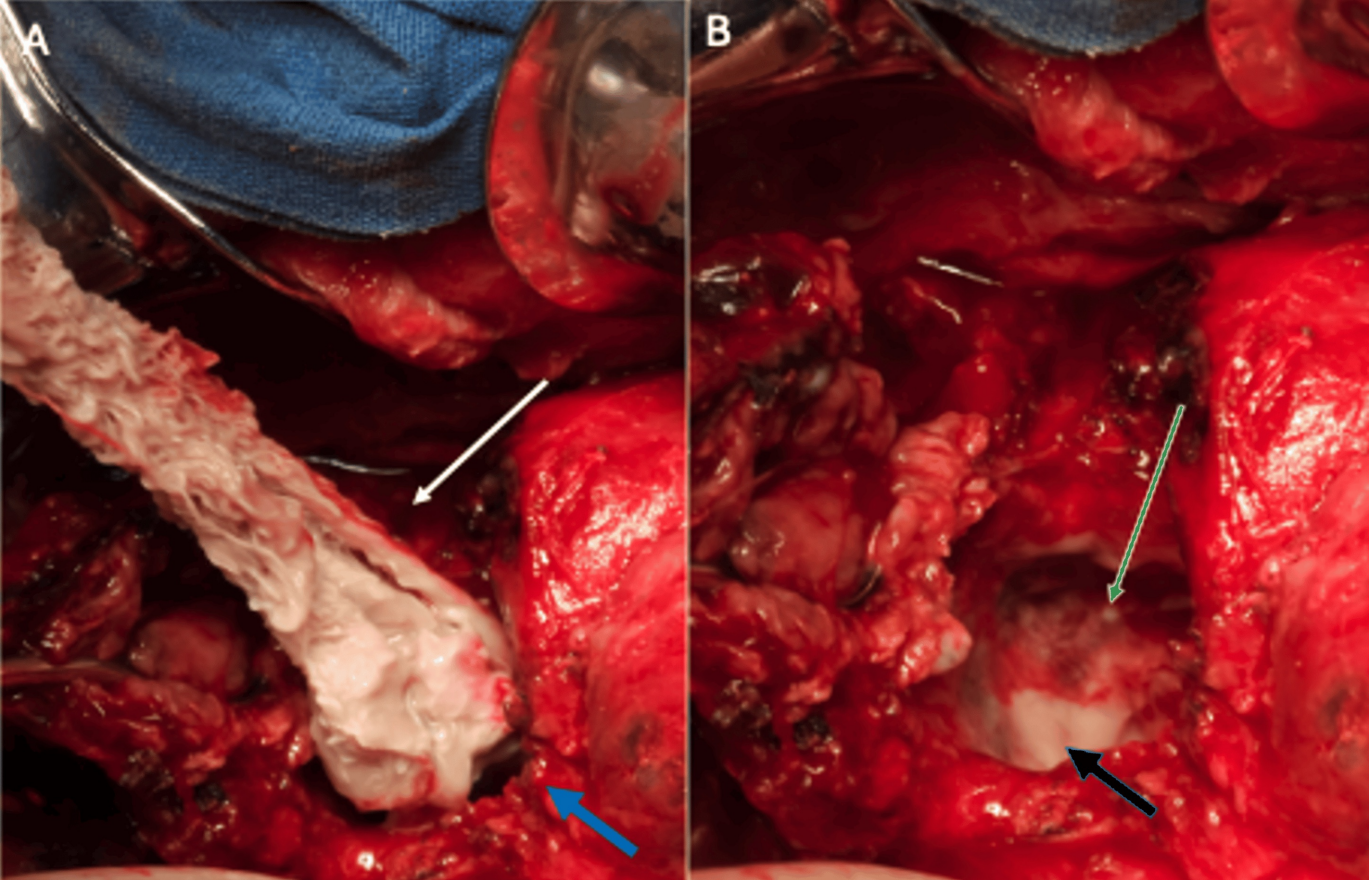
Intraoperative images. (A) An intraoperative view showing the extraction of a 3-cm intraparenchymal abscessed textile from segment V (white arrow), with the border of the liver (blue arrow). (B) An intraoperative view showing a cavity in segment V (green arrow). Purulent exudate at the base of the cavity is indicated by the black arrow at segment VIII. Original image by the authors.

Large abscesses in the liver, measuring 8 x 6 x 4 cm in segments VI and VII and a 6 x 6 cm abscess in segment V, were identified and drained. A sample was taken and sent for cultures. Upon draining and rupturing the abscess in segment V, an encased gossypiboma of approximately 3 cm was extracted (Figure 3). No bile duct injury was identified.

A Penrose drain was placed and directed toward the sub-hepatic space at the end of surgery. No evidence of bile leakage or disruption of the biliary tract was observed or documented.

During the postoperative period, the patient was kept on a levofloxacin-based antibiotic regimen for five days until culture results were available. The culture from the hepatic abscesses revealed *Escherichia coli*, which is sensible to imipenem. Consequently, the antibiotic regimen was switched to imipenem for 10 days; the susceptibility test is presented in Table 2.

**Table 2:** Antimicrobial susceptibility test

Antibiotic	MIC (Minimum Inhibitory Concentration)
Amikacin	<=2
Ampicillin/sulbactam	>=32
Cefepime	16
Cefoxitin	8
Ceftazidime	>=64
Ceftriaxone	>=64
Ciprofloxacin	>=4
Colistin	<=0.5
Doripenem	<=0.12
Ertapenem	<=0.5
Gentamicin	>=16
Imipenem	<=0.25
Meropenem	<=0.25
Piperacillin/tazobactam	<=4
Tigecycline	<=0.5
Detected microorganism	Escherichia coli

In the first 24 hours following surgery, the patient was started on a liquid diet, and the pain was managed with oral paracetamol 500 mg every eight hours and intravenous ketorolac 30 mg every eight hours as needed. During this period, the Penrose drain produced serosanguineous drainage. Over the next 24 hours, the patient did not experience nausea or vomiting and tolerated solid food well. In the following days, the drainage gradually decreased, allowing for the removal of the drain. The patient was subsequently discharged home with a follow-up appointment scheduled 10 days after discharge.

**Figure 4:**
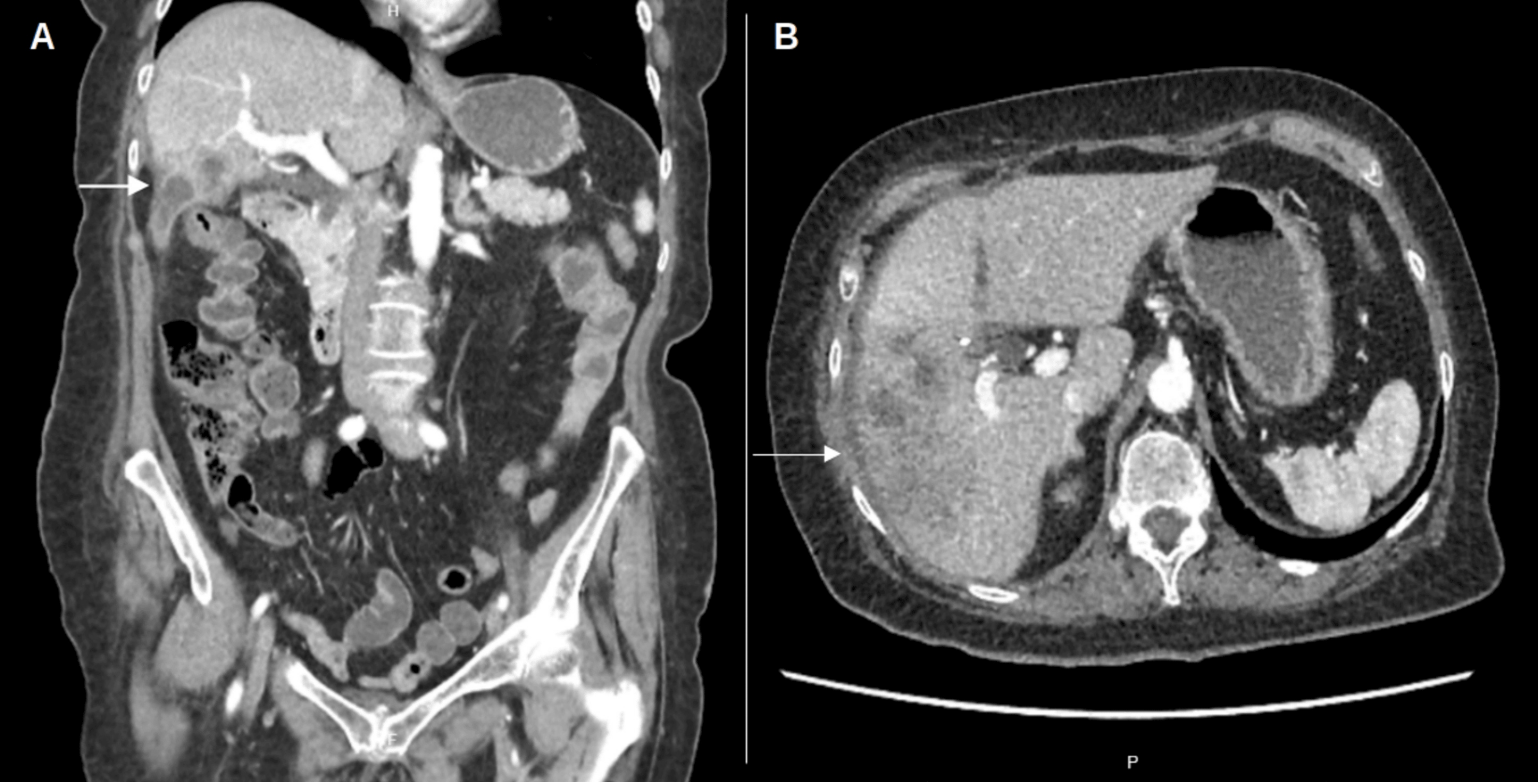
Follow-up abdominal CT scan. (A) Coronal view: hypodense areas are identified in segment V of the liver (white arrow). (B) Axial view: hypodense regions are observed in segments V and VI, consistent with an inflammatory process or residual abscess formation (white arrow). Original image by the authors.

Laboratory results during the initial outpatient appointment were within normal limits (Table 3). However, a contrast-enhanced abdominal CT scan revealed persistent small liver abscesses in segments V and VI, which extended into the right parietocolic gutter (Figure 4).

**Table 3:** Laboratory tests during follow-up Na: sodium; K: potassium; Cl: chloride; HCT: hematocrit; ALT: alanine aminotransferase; AST: aspartate aminotransferase; LDH: lactate dehydrogenase

Parameters	Admission	Follow-up	Normal Values
Glucose	108.7 mg/dL	105 mg/dL	70-105 mg/dL
Urea	17.3 mg/dL	17 mg/dL	15.2-52.3 mg/dL
Creatinine	0.6 mg/dL	0.74 mg/dL	0.57-1.11 mg/dL
Na	139 mEq/L	136 mEq/L	136-145 mEq/L
K	3.1 mEq/L	5 mEq/L	3.5-5.1 mEq/L
Cl	103.4 mEq/L	104 mEq/L	98-107 mEq/L
Hemoglobin	10 g/dL	11.7 g/dL	13-18 g/dL
HCT	29.8%	36.5%	42-53.6%
Platelets	334,000/mm^3^	422,000/mm^3^	150,000-450,000/mm^3^
Leukocytes	7600/mm^3^	6970/mm^3^	4600-10200/mm^3^
Total bilirubin	0.71 mg/dL	0.44 mg/dL	0.2-1.2 mg/dL
Direct bilirubin	0.4 mg/dL	0.23 mg/dL	0-0.5 mg/dL
Indirect bilirubin	0.27 mg/dL	0.21 mg/dL	0-0.8 mg/dL
ALT	30.6 IU/L	13 IU/L	0-55 IU/L
AST	94.8 IU/L	21 IU/L	5-34 IU/L
LDH	352 IU/L	192 IU/L	125-220 IU/L

The calculated volume of the abscesses was 70 cc. Given the patient's asymptomatic condition and clinical improvement, it was decided to proceed with oral treatment on an outpatient basis. She was prescribed oral metronidazole (500 mg every eight hours) and levofloxacin (500 mg once daily) for 14 days.

At the second postoperative appointment in August, two months after discharge, the patient remained asymptomatic. A follow-up abdominal ultrasound revealed no evidence of fluid collection or abnormalities in the liver. The pathology report identified a liver tissue fragment with acute abscessed inflammation, recent extensive hemorrhage, and chronic granulomatous inflammation featuring foreign body-type multinucleated giant cells. The patient remained under outpatient surveillance and has now completed one year of follow-up, during which she has remained asymptomatic.

## Discussion

Existing reports of intrahepatic gossypiboma primarily involve cases of retained foreign material after hepatic trauma; one of these reported cases was related to the non-absorption of gel foam following penetrating liver trauma, and another due to hepatic packing in a closed abdominal trauma [13,14]. Although Manzella et al. published a pictorial review showing an intrahepatic gossypiboma, no clinical data are presented to describe the case characteristics [15]. To the best of our knowledge, this is the first report of a gossypiboma fully encased in the hepatic parenchyma following non-trauma surgery.

In the available literature, several risk factors are associated with a higher likelihood of RSM, including emergency surgery, unexpected changes in the surgical procedure, higher BMI, blood loss greater than 500 cc, prolonged operative time, multiple sub-procedures, involvement of more than one surgical team, unexpected intraoperative findings, lack of surgical counts, and incorrect counts [6,16]. In our case, the complications after the index surgery and the related bleeding were the most likely cause of distraction, which led to the retention of surgical material inside the patient's abdomen. During the outpatient management, the patient reported mild fever, abdominal pain, and persistent purulent drainage until the foreign material was removed. The most reported symptoms associated with gossypiboma are pain, palpable mass, and fever [17]. It is plausible that the persistent drainage of the abdominal cavity limited the systemic reaction, enabling the patient to tolerate the foreign material for more than 19 months.

Reducing the incidence of RSM is a crucial goal within any healthcare system. Traditionally, manual counting has been the predominant method to detect missing surgical objects. However, a systematic review conducted by Weprin et al. revealed discrepancies in manual counting in approximately one out of every eight cases, with incorrect counts present in 62-88% of RSM events. X-rays have also been utilized to detect RSM, but they exhibit a false-negative rate of 10-30%, which varies depending on the size of the retained object. Emerging technologies such as RFID (radio frequency identification) sponge detection show promise in potentially reducing RSM incidence [6].

Nevertheless, until these new detection methods are widely available, adherence to rigorous security protocols in the operating room remains the primary approach to minimizing RSM occurrences.

## Conclusions

This report adds to the existing evidence of intrahepatic gossypibomas, highlighting how their identification can prevent treatment in non-specialized surgical centers and delay definitive surgical intervention. Gossypibomas continue to pose challenges across various healthcare systems and are often associated with the quality of security protocols established in hospitals, particularly in developing nations. The occurrence of these iatrogenic injuries highlights the need for stricter security protocols to be implemented and for new methods that can efficiently detect RSM to prevent harm to patients.
